# Mesenchymal Stromal Cells Adapt to Chronic Tendon Disease Environment with an Initial Reduction in Matrix Remodeling

**DOI:** 10.3390/ijms222312798

**Published:** 2021-11-26

**Authors:** Carla U. Doll, Sabine Niebert, Janina Burk

**Affiliations:** Equine Clinic (Surgery, Orthopedics), Justus-Liebig-University Giessen, Frankfurter Straße 108, 35390 Giessen, Germany; carla.u.doll@vetmed.uni-giessen.de (C.U.D.); sabine.niebert@vetmed.uni-giessen.de (S.N.)

**Keywords:** mesenchymal stromal cells (MSC), tendinopathy, chronic tendon disease, 3D cell culture, extracellular matrix, collagen, fibrosis, matrix remodeling, matrix–metalloproteinases (MMP), tenogenic differentiation

## Abstract

Tendon lesions are common sporting injuries in humans and horses alike. The healing process of acute tendon lesions frequently results in fibrosis and chronic disease. In horses, local mesenchymal stromal cell (MSC) injection is an accepted therapeutic strategy with positive influence on acute lesions. Concerning the use of MSCs in chronic tendon disease, data are scarce but suggest less therapeutic benefit. However, it has been shown that MSCs can have a positive effect on fibrotic tissue. Therefore, we aimed to elucidate the interplay of MSCs and healthy or chronically diseased tendon matrix. Equine MSCs were cultured either as cell aggregates or on scaffolds from healthy or diseased equine tendons. Higher expression of tendon-related matrix genes and tissue inhibitors of metalloproteinases (TIMPs) was found in aggregate cultures. However, the tenogenic transcription factor scleraxis was upregulated on healthy and diseased tendon scaffolds. Matrix metalloproteinase (MMPs) expression and activity were highest in healthy scaffold cultures but showed a strong transient decrease in diseased scaffold cultures. The release of glycosaminoglycan and collagen was also higher in scaffold cultures, even more so in those with tendon disease. This study points to an early suppression of MSC matrix remodeling activity by diseased tendon matrix, while tenogenic differentiation remained unaffected.

## 1. Introduction

Tendon disease is a common reason for early retirement from athletic activities, as their incidence increases with age and exercise level [1,2]. Tendons heal slowly and with formation of scar tissue. Once an acute tendon injury has occurred, the healing process begins with a short inflammatory phase of a few days up to 2 weeks. Even during this early phase and continuing successively, connective tissue is formed under vascular sprouting and cellular infiltration, showing increased levels of type III collagen and altered concentrations of glycosaminoglycans. Histologically, there are also changes in the matrix structure, such as a decrease in the density of collagen fibers and a loss of their parallel arrangement [3]. The excessive and immature formation of extracellular matrix by fibroblasts and myofibroblasts is referred to as fibrosis, the main mediator of which is considered to be transforming growth factor (TGF)-β1 [3,4]. Over time, matrix composition improves as collagen III is degraded and replaced by collagen I, which is controlled by the interplay of enzymes. These include matrix metalloproteinases (MMPs) and their inhibitors, the tissue inhibitors of metalloproteinases (TIMPs) [5,6]. Nevertheless, the composition and fiber organization within the tendon remain altered, and the newly formed scar tissue can achieve the strength but not the elasticity of healthy tendon tissue. This often leads to re-injuries, and chronic disease progresses when training is resumed [3].

To prevent the chronic development of the disease, improved regenerative therapies for tendon disease are necessary. Tendon disease is not only observed in human athletes but also in horses. In racehorses, the superficial digital flexor tendon of the forelimb is most commonly affected, accounting for 89% of all tendon and ligament injuries [7]. The function and clinical relevance of this tendon is comparable to the Achilles tendon in humans, making it an excellent natural disease model for Achilles tendon pathology in humans. In horses, the local injection of multipotent mesenchymal stromal cells (MSCs) has been repeatedly reported to improve tendon healing after acute injury, leading to reduced recurrence rates [8,9,10,11,12]. This success is primarily attributed to a stimulation of neovascularization as well as to anti-apoptotic and immunomodulatory effects [13]. However, so far, existing data suggest that treatment might be less successful in chronic tendon disease [12]. In the latter, the extracellular matrix pathology is predominant; thus, it would be favorable to make use of the anti-fibrotic properties of MSCs. 

Transplantation of MSCs was already shown to significantly reduce fibrosis in the tissues of the heart, lung, liver, kidney, and cornea [14]. In an experiment with rats, it was shown that intravenous administration of MSCs could reduce the expression of fibrosis factors such as α-smooth muscle actin and TGF-β1 [15]. Moreover, in fibrocytes, mRNA expression of collagen III, TGF-ß1 receptor and smad3 was shown to be decreased in the presence of MSCs [16]. In addition to their paracrine effects that may indirectly support matrix remodeling, MSCs also have a direct effect on the extracellular matrix as they can secrete TIMPs and MMPs and activate exogenous pro-MMPs [17,18]. However, in contrast to the immunomodulatory mechanisms of MSCs, their matrix-modulating properties have been addressed very little so far. In particular, it is not known how pre-existing changes in the extracellular matrix would affect MSCs. Therefore, the current knowledge does not provide a sufficient basis for the use of MSCs in chronic tendon disease yet. 

Aiming to elucidate the potential of MSCs to treat chronic tendon disease, in the present study, the tenogenic and matrix-modulatory behavior of MSCs was investigated using an ex vivo model of equine naturally occurring chronic tendon disease. In the first set of experiments, we compared MSCs cultured on scaffolds obtained from a healthy tendon to MSCs cultured in a three-dimensional (3D) aggregate culture. In the second part of the study, MSCs were cultured on scaffolds obtained from equine tendons with previously characterized chronic disease, as compared to scaffolds from healthy tendons. We hypothesized that the tendon extracellular matrix would affect MSC behavior and particularly their matrix remodeling properties.

## 2. Results

### 2.1. MSC Culture as Aggregates and on Healthy Tendon Scaffolds

After culturing MSCs from *n* = 6 donor horses either as cell aggregates or on decellularized healthy equine tendon scaffolds from the same donor horse for 3 and 6 days, quantitative RT-PCR and supernatant analyses revealed how the healthy tendon extracellular matrix environment influenced MSC behavior as compared to a scaffold-free 3D environment.

#### 2.1.1. Tendon Marker, Growth Factor, MMP, and TIMP Gene Expression

**Figure 1:**
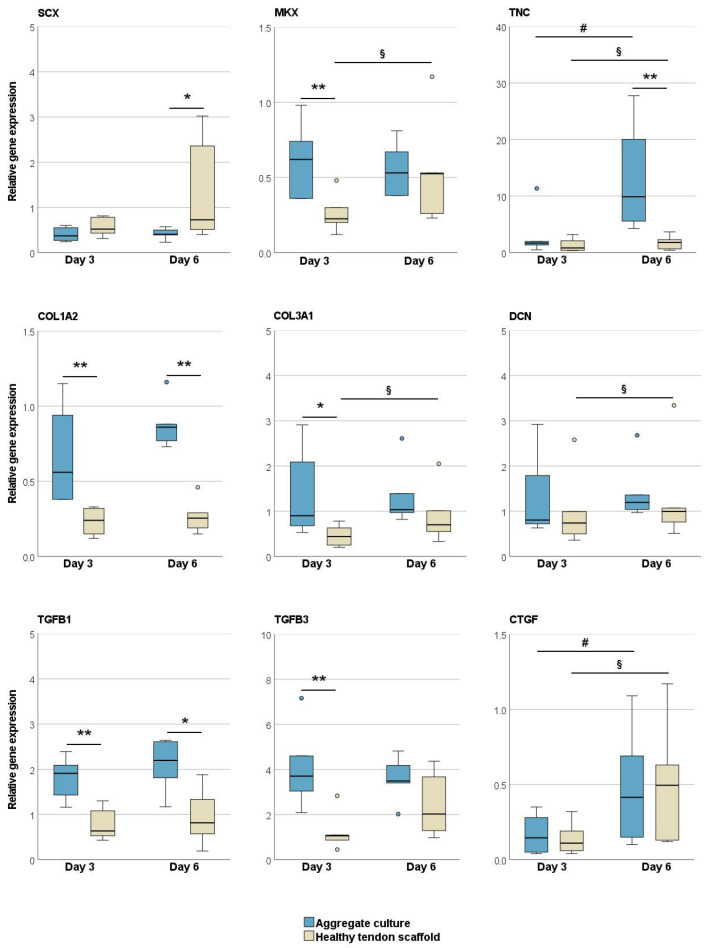
Relative gene expression of tendon-related genes and growth factors in MSC aggregate cultures and healthy tendon scaffold cultures. Asterisks indicate significant differences between groups with *p* < 0.05 (*) or *p* < 0.01 (**). Significant differences over time are indicated by hashmarks for aggregate cultures and by paragraph symbols for healthy tendon scaffold cultures (*p* < 0.05). Data were obtained with MSCs from *n* = 6 donors. SCX: scleraxis; MKX: mohawk; TNC: tenascin-C; COL1A2: collagen I; COL3A1: collagen III; DCN: decorin; TGFB: transforming growth factor-β; CTGF: connective tissue growth factor.

The tenogenic transcription factor scleraxis was upregulated in the MSCs in healthy tendon scaffold cultures as compared to aggregate cultures (*p* < 0.05 at day 6). All other tendon-related genes, including the late tenogenic transcription factor mohawk, tendon extracellular matrix components, and tenogenic and pro-fibrotic growth factors, were expressed at higher levels in the MSC aggregate cultures. This was significant on both day 3 and day 6 for collagen I (*p* < 0.01) and TGF-β1 (*p* < 0.01 and *p* < 0.05), on day 3 for mohawk, TGF-β3 (*p* < 0.01) and collagen III (*p* < 0.05), and on day 6 for tenascin-C (*p* < 0.01). An increase in gene expression over time, although partly with low fold changes, was observed in the aggregate cultures for tenascin-C and connective tissue growth factor (CTGF) and in the healthy tendon scaffold cultures for collagen III, tenascin-C, decorin, and CTGF (*p* < 0.05 for all) (Figure 1).

**Figure 2:**
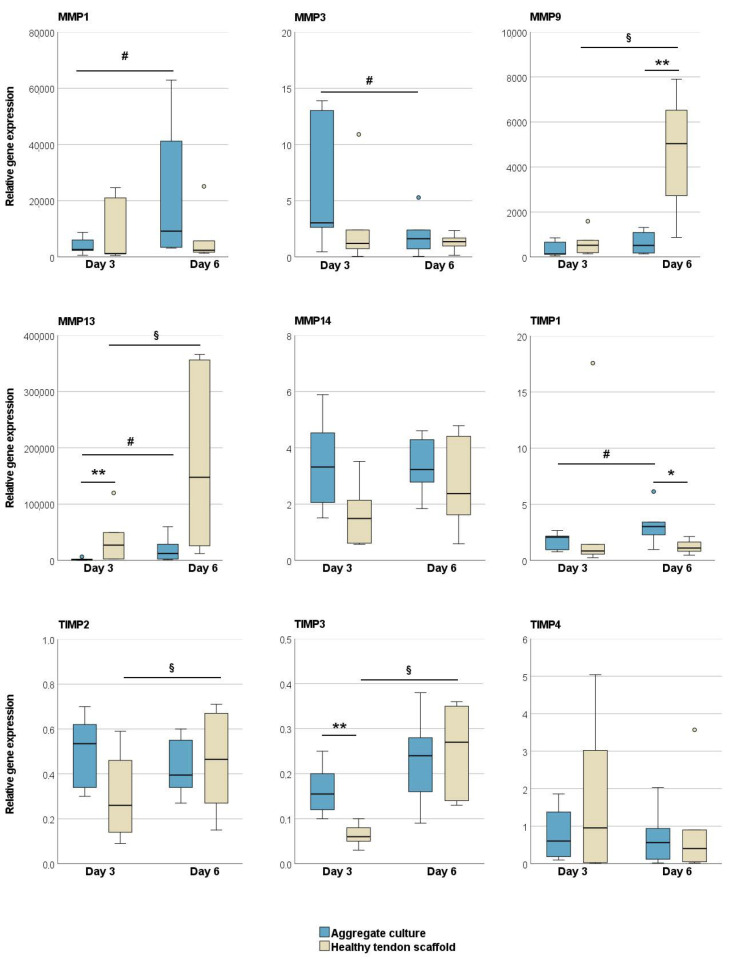
Relative gene expression of matrix metalloproteinases (MMPs) and tissue inhibitors of metalloproteinases (TIMPs) in MSC aggregate cultures and healthy tendon scaffold cultures. Asterisks indicate significant differences between groups with *p* < 0.05 (*) or *p* < 0.01 (**). Significant differences over time are indicated by hashmarks for aggregate cultures and by paragraph symbols for healthy tendon scaffold cultures (*p* < 0.05). Data were obtained with MSCs from *n* = 6 donors.

Regarding MMPs and TIMPs, there was a trend that, overall, MMPs were expressed at higher levels in the tendon scaffold cultures, but TIMPs in the aggregate cultures. When cultured on healthy tendon scaffolds, MSCs showed a higher expression of MMP13 (*p* < 0.01 on day 3) and MMP9 (*p* < 0.01 on day 6) as compared to aggregate cultures, with an additional increase over time (*p* < 0.05). An increase over time in tendon scaffold cultures was also observed for TIMP-2 and -3 expression (*p* < 0.05). On the other hand, the expression of TIMP3 on day 3 (*p* < 0.01) and TIMP1 on day 6 (*p* < 0.05) was higher in MSC aggregate cultures than in tendon scaffold cultures. Furthermore, in MSC aggregate cultures, there was an increase in MMP1 and TIMP1 but a decrease in MMP3 expression over time (*p* < 0.05 for all) (Figure 2).

#### 2.1.2. Glycosaminoglycan and Total Collagen Release and MMP Activity

**Figure 3:**
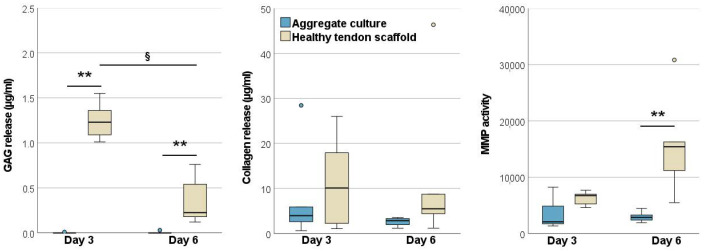
Glycosaminoglycan, total collagen and matrix metalloproteinase (MMP) activity in the supernatants of MSC aggregate cultures and healthy tendon scaffold cultures. Asterisks indicate significant differences between groups with *p* < 0.01 (**). Significant differences over time are indicated by paragraph symbols for healthy tendon scaffold cultures (*p* < 0.05). Data were obtained with MSCs from *n* = 6 donors.

In the healthy tendon scaffold cultures, more glycosaminoglycan and total collagen were released into the supernatant than in the aggregate cultures, yet this was only significant for glycosaminoglycan (*p* < 0.01 on both day 3 and day 6). However, the glycosaminoglycan release from tendon scaffold cultures decreased from day 3 to day 6 (*p* < 0.05). The activity of MMPs in the supernatants was higher in tendon scaffold cultures (*p* < 0.01 on day 6) (Figure 3).

### 2.2. MSC Culture on Healthy and Chronic Tendon Disease Scaffolds

MSCs from one representative donor horse, as chosen based on the results described above, were cultured either on decellularized healthy tendon scaffolds or on decellularized tendon scaffolds with chronic disease from *n* = 5 donor horses per group for 3, 6, or 21 days. Morphological analyses, quantitative RT-PCR and supernatant analyses demonstrated how the chronically altered tendon matrix influenced MSC behavior. 

#### 2.2.1. Morphology of Scaffolds and Scaffold Cultures

**Figure 4:**
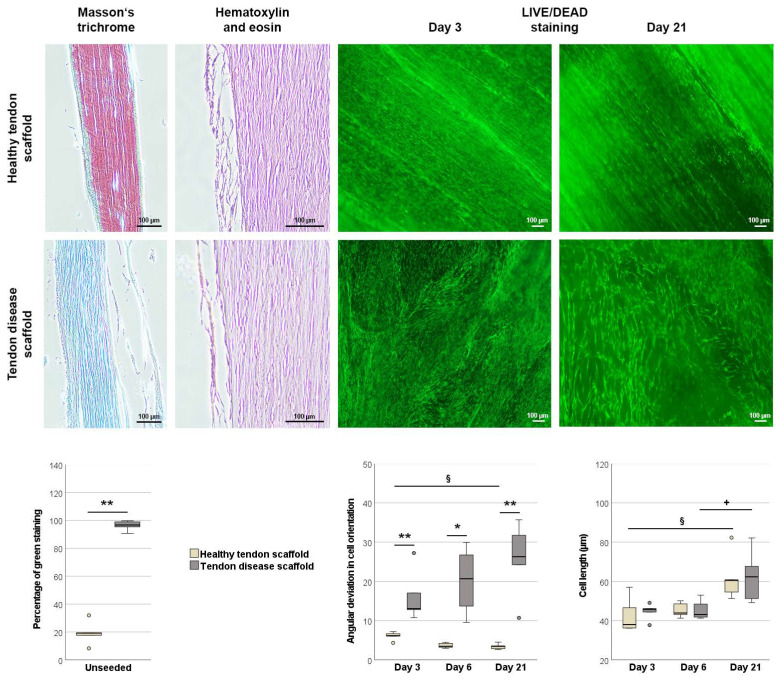
Morphological analysis of unseeded and MSC-seeded healthy and diseased tendon scaffolds. Masson’s trichrome staining was performed to confirm tendon disease using unseeded scaffolds. Healthy scaffolds stained mainly red, representing healthy tendon fibers, while tendon disease scaffolds stained mainly green, representing altered tendon tissue. The boxplot displays the percentage of green staining. Hematoxylin and eosin staining of seeded tendon scaffolds showed that cell layers were mostly found on the scaffold surface, with no major differences between the scaffold groups. Live/Dead staining (live cells shown in green) of tendon scaffold cultures revealed a more random orientation of MSCs on the disease scaffolds, as quantified by the mean angular deviation from the median angle in cell orientation shown in the boxplot. It also showed that MSC length increased over time, which was also quantitatively confirmed as shown in the boxplot. All scale bars: 100 µm. Asterisks indicate significant differences between groups with *p* < 0.05 (*) or *p* < 0.01 (**). Significant differences over time are indicated by paragraph symbols for healthy tendon scaffold cultures and by plus symbols for diseased tendon scaffold cultures (*p* < 0.05). Data were obtained using scaffolds from *n* = 5 different tendons per group.

Healthy tendon scaffolds and those with chronic disease, as previously characterized by magnetic resonance imaging, strongly differed in Masson’s trichrome staining of their paraffin sections (*p* < 0.01). Confirming their designation to the healthy and to the chronic disease group, the extracellular matrix in the healthy scaffolds mainly stained red, whereas diseased scaffolds mainly stained green (Figure 4). 

Hematoxylin and eosin staining of paraffin sections of the seeded scaffolds demonstrated that MSCs were mostly localized on the scaffold surface, with no significant differences in score points between groups or over time (data not shown). However, quantitative analysis of Live/Dead staining images revealed remarkable differences between groups. Most importantly, while MSCs were aligned in widely parallel rows on the healthy tendon scaffolds, they were randomly oriented on tendon disease scaffolds. This was confirmed by a higher angular deviation of cell orientation in tendon disease scaffolds at all time points (*p* < 0.01 on days 3 and 21, *p* < 0.05 on day 6). Over time, the deviation in cell orientation increased further on tendon disease scaffolds and decreased further on healthy scaffolds (*p* < 0.05 for the latter). MSC length increased over time in both groups (*p* < 0.05) but did not differ between groups (Figure 4).

#### 2.2.2. Tendon Marker, Growth Factor, MMP, and TIMP Gene Expression

**Figure 5:**
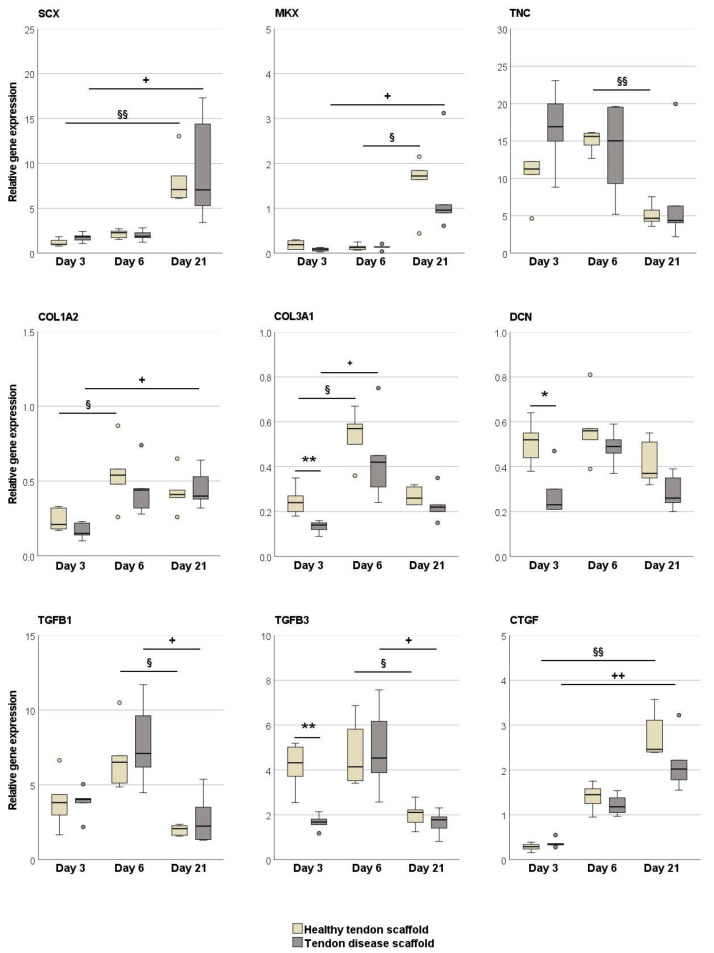
Relative gene expression of tendon-related genes and growth factors in MSCs seeded on healthy and diseased tendon scaffolds. Asterisks indicate significant differences between groups with *p* < 0.05 (*) or *p* < 0.01 (**). Significant differences over time are indicated by paragraph symbols for healthy tendon scaffold cultures and by plus symbols for diseased tendon scaffold cultures (§ or + for *p* < 0.05; §§ or ++ for *p* < 0.01). Data were obtained using scaffolds from *n* = 5 different tendons per group. SCX: scleraxis; MKX: mohawk; TNC: tenascin-C; COL1A2: collagen I; COL3A1: collagen III; DCN: decorin; TGFB: transforming growth factor-β; CTGF: connective tissue growth factor.

The tenogenic transcription factors scleraxis and mohawk were upregulated over time (*p* < 0.01 for scleraxis in MSCs on healthy tendon scaffolds, *p* < 0.05 for all other comparisons). However, interestingly, with respect to MSC tenogenic induction, there appeared to be no differences between the healthy and diseased tendon groups. In line with that, collagen I was also upregulated over time in both groups (*p* < 0.05) and with no differences between groups. Other matrix components, which are less abundant in healthy tendon tissue, were only temporarily upregulated but then downregulated over time. However, this was only significant for the collagen III upregulation between day 3 and day 6 (*p* < 0.05) and for the downregulation of tenascin-C between days 6 and 21 in MSCs on healthy scaffolds (*p* < 0.01). TGF-β1 and -3 showed a similar regulation pattern, with a decrease in expression between day 6 and day 21 (*p* < 0.05), whereas CTGF showed a consistently increasing expression between day 3 and day 21 (*p* < 0.01). Differences between groups only occurred on day 3, with a lower expression of collagen III and TGF-β3 (both *p* < 0.01) and decorin (*p* < 0.05) in the MSCs cultured on chronic disease tendon scaffolds (Figure 5).

**Figure 6:**
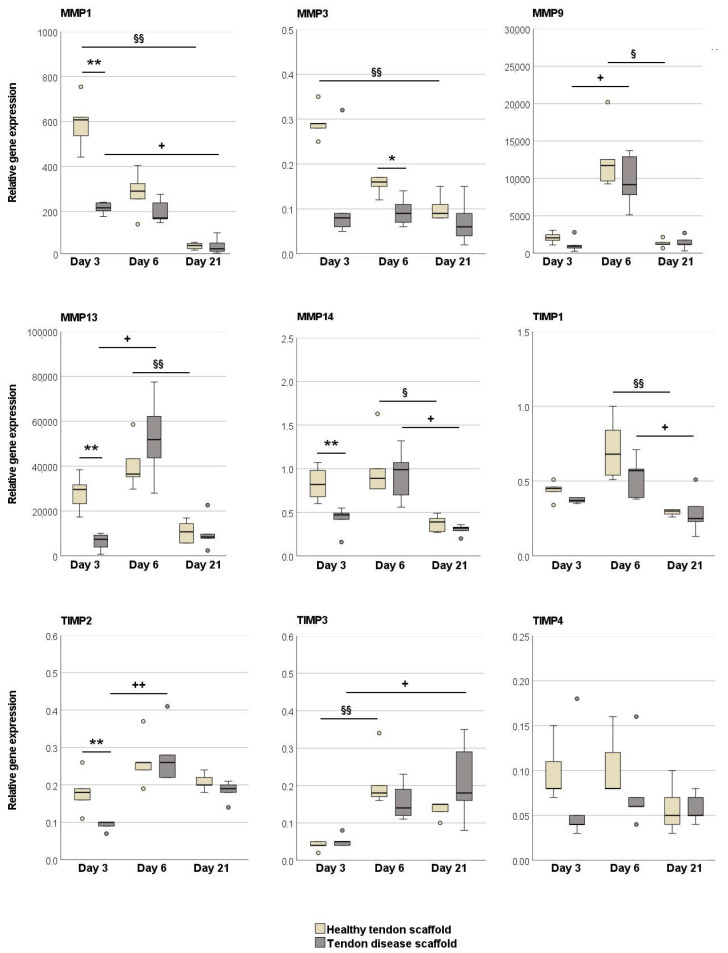
Relative gene expression of matrix metalloproteinases (MMPs) and tissue inhibitors of metalloproteinases (TIMPs) in MSCs seeded on healthy and diseased tendon scaffolds. Asterisks indicate significant differences between groups with *p* < 0.05 (*) or *p* < 0.01 (**). Significant differences over time are indicated by paragraph symbols for healthy tendon scaffold cultures and by plus symbols for diseased tendon scaffold cultures (§ or + for *p* < 0.05; §§ or ++ for *p* < 0.01). Data were obtained using scaffolds from *n* = 5 different tendons per group.

MMPs were either downregulated over time (*p* < 0.01 on healthy scaffolds for MMP1 and MMP3, *p* < 0.05 on disease scaffolds for MMP1) or showed a temporal regulation with the highest expression levels on day 6 (*p* < 0.05 or *p* < 0.01 when compared to earlier or later time points in both groups for MMP9, MMP13, and MMP14). A similar regulation over time, with the highest expression on day 6, was observed for TIMP1 and TIMP2 (*p* < 0.05 or *p* < 0.01 when compared to day 21 for TIMP1 in both groups, *p* < 0.01 when compared to day 3 for TIMP2 in MSCs on healthy tendon scaffolds). TIMP3 was upregulated over time (*p* < 0.01 for MSCs on healthy scaffolds between day 3 and 6, *p* < 0.05 for MSCs on chronic disease scaffolds between day 3 and 21). Importantly, there were significant differences between the groups, and these consistently involved lower gene expression levels in MSCs cultured on tendon disease scaffolds, which was most evident on day 3. Namely, MMP1, MMP13, and MMP14 but also TIMP2 were downregulated on diseased scaffolds on day 3 (*p* < 0.01 for all), and MMP3 on day 6 (*p* < 0.05) (Figure 6).

#### 2.2.3. Glycosaminoglycan and Total Collagen Release and MMP Activity

**Figure 7:**
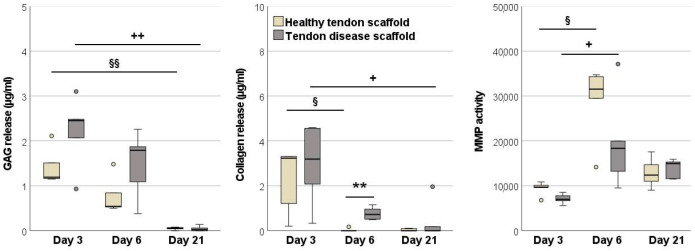
Glycosaminoglycan, total collagen, and matrix metalloproteinase (MMP) activity in the supernatants of healthy and diseased tendon scaffold cultures. Asterisks indicate significant differences between groups with *p* < 0.01 (**). Significant differences over time are indicated by paragraph symbols for healthy tendon scaffold cultures and by plus symbols for diseased tendon scaffold cultures (§ or + for *p* < 0.05; §§ or ++ for *p* < 0.01). Data were obtained using scaffolds from *n* = 5 different tendons per group.

In the tendon disease scaffold cultures, glycosaminoglycan and total collagen release into the supernatants were higher than in healthy tendon scaffold cultures, although this was only significant for total collagen on day 6 (*p* < 0.01). Remarkably, the release of both matrix components strongly decreased over time in both groups (*p* < 0.01 for glycosaminoglycans between days 3 and 21, *p* < 0.05 for collagen between days 3 and 6 in healthy scaffold cultures and between days 3 and 21 in disease scaffold cultures) and was at a neglectable level by day 21. Despite the higher release of matrix components, but still mirroring the gene expression results, MMP activity in the supernatant was highest on day 6 in both groups (*p* < 0.05 as compared to day 3). Moreover, MMP activity was higher in the healthy scaffold cultures than in the disease scaffold cultures on day 3 and 6, yet this failed to reach significance (Figure 7).

## 3. Discussion

In this study, we aim to elucidate the potential performance of MSCs injected into a chronic tendon lesion using an ex vivo model for naturally occurred chronic tendon disease. We focused on the matrix remodeling effects of the MSCs, as they likely play a major role when MSCs are used for treatment of an already fibrotic tendon.

The model we chose was unique in that decellularized extracellular matrices obtained from actual chronic tendon disease served as scaffolds for MSC culture. The preparation of decellularized tendon scaffolds and their seeding with MSCs is well established and mirrors the extracellular tendon environment better than artificial scaffolds [19,20,21]. Moreover, with respect to investigating tendon disease, the changes to the tendon extracellular matrix in chronic disease are complex [22] and cannot be reflected completely by any artificial scaffold material. Therefore, we chose to harvest tendons from an abattoir and screen them macroscopically and by magnetic resonance imaging. For the latter, we used procedures described previously [23] to distinguish acute and chronic lesions. Suitable tendons were then used to produce either healthy or chronic disease scaffolds, depending on the magnetic resonance imaging findings, and health or disease of the scaffold extracellular matrix was confirmed by histology. It remains a limitation that the exact lesion age was unknown due to the lacking clinical history of the donor horses, yet based on MRI and histology findings, it is without question that chronic lesions were present in all disease group scaffolds and absent in all healthy scaffolds.

First, we aimed to characterize MSC tenogenic differentiation and matrix remodeling on healthy tendon scaffolds. As scaffold seeding is a 3D culture system, we included a different, frequently used 3D culture system, namely scaffold-free aggregate cultures, as a control condition. In the literature, 3D aggregate cultures are described using highly variable cell numbers and experiment settings [24,25,26,27,28,29]. In this study, the aggregate culture conditions were kept as similar as possible to the scaffold culture conditions, which means that the same relation between cell numbers and medium volumes was used, and no dynamic stimulation was applied. Interestingly, while the early tenogenic marker scleraxis was upregulated on healthy scaffolds as compared to the aggregates, other tendon and matrix genes, as well as TGF-β1 and -β3, were expressed at higher levels in the aggregate cultures. This is in accordance with the overall growing knowledge regarding increased MSC fitness after 3D aggregate or spheroid culture. Pre-culturing MSCs as spheroids appears to have positive effects on their regenerative potential [30,31]. Corresponding studies have already been performed for wound healing, treatment of the central nervous system, and myocardial infarction, with improved anti-fibrotic effects of the MSCs [32,33,34,35]. With respect to tendon, others reported that 3D spheroid culturing maintained the tenogenic [29,36] or ligamentogenic [37] differentiation in contrast to monolayer culture. Co-culture with tenocytes or culture with spent medium from tenocytes in high-density cultures further promoted tenogenic effects [38]. On that basis, MSC aggregate or spheroid cultures represent a promising starting material for scaffold seeding and tissue engineering [25,39,40,41]. Nevertheless, as addressed below, aggregate culture did not stimulate MSC matrix remodeling activities.

Next, comparing MSCs cultured on healthy and chronic disease tendon scaffolds, the first remarkable observation was that MSC alignment on the scaffolds clearly reflected the altered tendon fiber orientation in chronic disease. We observed not only a parallel MSC alignment in healthy scaffolds, as described before [19], but also a recurrent random alignment in the disease group. However, interestingly, although it is known that scaffold architecture directs differentiation [42,43], there was a similar upregulation of both tenogenic transcription factors, scleraxis and mohawk, over time in both groups. This suggests that, fortunately, the tenogenic potential of MSCs is not affected by the extracellular tendon matrix altered by chronic disease. Further supporting this, collagen I expression and MSC length also increased over time in both groups. In this line, it should also be noted that CTGF was similarly upregulated over time in both groups. The consequences are unequivocal, as CTGF has been associated with tendon healing [44,45] but is also strongly implicated in fibrosis [46]. Regarding extracellular matrix gene expression, collagen III and decorin expression was lower on tendon disease scaffolds. Both are essential but not very abundant components in healthy tendons. As collagen III is increased in tendon lesions [47], it could be hypothesized, although speculatively, that collagen III downregulation in MSCs on disease scaffolds could be a valuable feedback mechanism to their pathological abundance in the scaffold. Decorin concentration does not seem to be affected in tendon lesions [48]. Nevertheless, in our study, its expression was downregulated in tendon disease scaffolds. Based on its inhibiting effects on the pro-fibrotic growth factor TGF-β1 [49], decorin might have scar-preventing effects. On the other hand, it is involved in collagen fibrillogenesis and is associated with a weakening of tendons as it leads to a decrease in collagen fiber diameter [50]. However, the expression of TGF-β3 was also lower in the tendon disease group. This could rather be a drawback as TGF-β3 is known for its positive effects on tenogenesis [51,52], yet this difference was only evident after a short period of culture (day 3).

Intriguingly, the matrix remodeling activities of MSCs strongly changed in response to their extracellular environment. This was not unexpected as MMP expression has already been reported to be influenced by extracellular matrix components and stiffness [53,54]. First of all, the expression of MMP1, MMP9, and MMP13 was strongly upregulated in all groups compared to the monolayer controls (data used for Pfaffl calculation), leading to very high fold changes in expression values [55]. Moreover, several differences were observed between the different 3D cultures investigated. Overall, the gene expression of several MMP was highest in MSCs cultured on healthy tendon scaffolds. The expression levels of the gelatinase MMP9 and the collagenase MMP13 were higher in healthy scaffold cultures than in aggregate cultures, while TIMP1 and TIMP3 expression was lower, suggesting a higher matrix remodeling activity in the healthy scaffold cultures. This was confirmed by a higher release of matrix components into the supernatant and a higher total MMP activity in the supernatant. Tendon disease reduced the matrix remodeling activity of MSCs. The expression levels of the collagenases MMP1 and MMP13, the stromelysin MMP3, and the membrane-type collagenase MMP14, as well as the total MMP activity in the supernatant, were lower in MSCs cultured on disease tendon scaffolds as compared to healthy tendon scaffolds. Interestingly, these differences occurred early during culture, while MMP gene expression and activity in both groups tended to change over time. The initially reduced MMP activity in the disease group is consistent with an own previous study, which had revealed a reduced MMP activity when MSCs were cultured on pro-fibrotic MSC-derived matrices [56]. However, surprisingly, in the current study, the release of extracellular matrix components into the supernatants was even higher in disease scaffold cultures, possibly because the structure of tendon disease scaffolds is generally more prone to degradation.

The implication of the putatively reduced matrix remodeling activity of MSCs in chronic tendon disease is not readily explainable. Data on matrix remodeling in tendons are still not entirely conclusive, considering the complex interplay between MMPs, TIMPs, growth factors and the extracellular matrix. In vivo, increased MMP expression is often associated with tendon disease [57,58,59] and attempts have been made to inhibit MMP in order to prevent the progression of tendon disease [60,61]. In acute tendon (rupture) repair, several MMPs, including MMP1, MMP2, MMP9, and MMP13, were upregulated [57,62,63], while MMP3 expression was reported to be lower [5] or to decrease over time [57]. Low MMP3 and MMP10 expression levels were also associated with chronic painful tendon disease [5]. Yet, in a different study, MMP3 expression correlated with neoangiogenesis and impaired biomechanical properties during tendon healing [64]. In horses with tendon disease, which had been treated by local MSC transplantation, a lower MMP13 activity in the MSC-treated group was associated with a better histological appearance and mechanical properties 6 months after treatment [65]. In another equine study, MMP3 gene expression was upregulated in the MSC-treated tendons at week 45 [66]. Based on most of these findings taken together and considering tendon pathophysiology, it might be concluded that low expression and activity of collagenases such as MMP13 could prevent further degradation, while a high expression and activity of MMPs that degrade smaller matrix components, such as MMP3, could be helpful for removal of increased non-fibrillar components. On the other hand, it could even be speculated that in chronic tendon disease, MMPs might generally play a supportive role, as the fibrotic scar tissue has to be degraded to be finally replaced by tendon tissue of a higher quality.

## 4. Materials and Methods

### 4.1. Study Design

**Figure 8:**
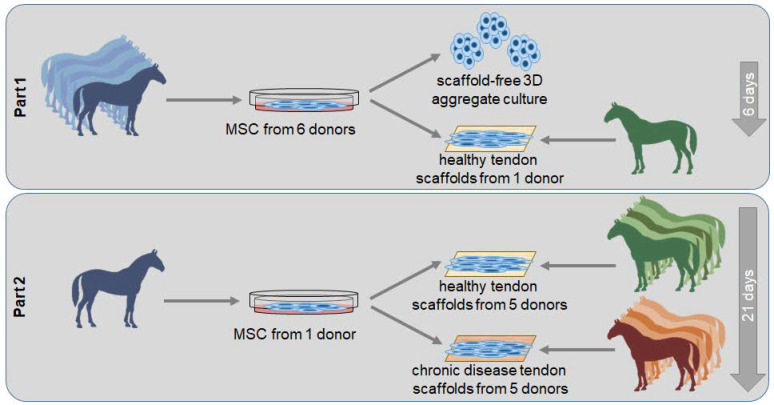
Overview of the experimental design.

The study included two sets of experiments (Figure 8). In the first part, aiming to investigate the effect of healthy extracellular matrix on MSC tenogenic and matrix remodeling behavior, MSCs from 6 equine donors were used as biological replicates. They were cultured on decellularized healthy tendon scaffolds, all obtained from the same tendon, while aggregate cultures served as 3D control condition. Gene expression and cell culture supernatants were analyzed on day 3 and day 6. The second part aimed to investigate the effect of chronic tendon disease, namely of the altered extracellular matrix, on MSC tenogenic and matrix remodeling behavior. For this purpose, MSCs from the donor horse best representing the median results from the first part were used for all experiments, while the tendons used for scaffold production represented the biological replicates. MSCs were cultured on healthy tendon scaffolds, obtained from 5 different healthy tendons, and on tendon disease scaffolds, obtained from 5 different tendons with naturally occurring chronic tendon disease. Morphology, gene expression and cell culture supernatants were analyzed on day 3, day 6, and day 21.

### 4.2. Tendon Scaffold Preparation

Superficial digital flexor tendons were dissected from equine forelimbs obtained from an abattoir. Based on macroscopic and ultrasonographic evaluation, tendons were used in a comparative study on tendon lesion appearance in low-field magnetic resonance imaging (Hallmarq EQ2, Hallmarq Veterinary Imaging, Guildford, Surrey, UK) [23] and high-field magnetic resonance imaging (Magnetom Verio; Siemens Healthcare GmbH, Erlangen, Germany) (unpublished data). Based on these data, healthy tendons and tendons with naturally occurred chronic tendon disease were selected for the current study.

Tendons were decellularized by combining freeze–thaw cycles and detergent treatment as established previously [67]. After decellularization, tendons were stored at −20 °C until the scaffolds were sectioned. First, 1 cm of the length of the tendon was excised, taking care to cover only the lesion region in the case of the diseased tendons. Next, these tendon pieces were cut into 300 µm thick scaffolds using a cryotome (Leica CM3050 S, Wetzlar, Germany). The obtained scaffolds were finally trimmed to a surface area of 1 × 1 cm. 

### 4.3. Mesenchymal Stromal Cell Isolation

Subcutaneous supragluteal adipose tissue was obtained from 6 warmblood horses aged 1–6 years that were euthanized for reasons unrelated to this study. Mesenchymal stromal cells were isolated by mechanical comminution and following collagenase I (0.8 mg/mL; Life Technologies, Karlsruhe, Germany) digestion. The obtained MSCs were expanded and cryopreserved. MSCs from the most representative donor, which were used for both parts of the study, were characterized by trilineage differentiation and flow cytometry (Supplement S1).

### 4.4. Cell Culture Experiments

MSCs were thawed and expanded in Dulbecco’s modified Eagle’s medium (1 g/L glucose; Gibco^®^, ThermoFisher Scientific, Darmstadt, Germany) supplemented with 10% FBS (Gibco^®^) and 1% penicillin-streptomycin (Pan-Biotech, Aidenbach, Germany) under standard culture conditions (humidified atmosphere, 37 °C, 5% CO_2_) until passage 3. For the formation of aggregates, 900,000 MSCs were seeded in 1,500 µL medium in a 24-well ultra-low attachment plate. Scaffolds were placed in a 24 well ultra-low attachment plate, seeded with 300,000 MSCs per scaffold and incubated with 500 µL medium after an initial period allowing for cell attachment (6 h). Medium was changed every 3 days, and the supernatants were stored at −80 °C. For medium changes, aggregates were centrifuged at 300× *g* for 3 min to separate them from the supernatant.

### 4.5. Histology

Unseeded and seeded scaffolds were fixed in 4% paraformaldehyde and embedded standing upright in paraffin. Four-micrometer-thick longitudinal sections of the unseeded scaffolds were subjected to Masson’s Trichrome staining, while sections of the seeded scaffolds were stained with hematoxylin and eosin (HE). For Masson’s trichrome stained scaffolds, three images were obtained with a 10× objective, and the percentage of green staining, displaying altered tendon tissue, was quantified using Fiji/ImageJ (National Institutes of Health, Bethesda, MD, USA) software. HE stained seeded scaffolds were scored by two observers with respect to cell distribution on and within the scaffold. 

### 4.6. Live/Dead Staining

Live/Dead staining was performed using a staining kit (LIVE/DEAD™ Viability/Cytotoxicity Kit, for mammalian cells; Thermo Fisher Scientific, Waltham, MA, USA) according to the manufacturer’s instructions. One image using the 4× objective, representing the middle of the scaffold, and 3 images using the 10× objective, evenly distributed on one diagonal of the scaffold, were taken using a Nikon Eclipse Ts2-F microscope with a DS-Fi3 camera (Nikon GmbH, Duesseldorf, Germany). 

Images obtained from the middle of the scaffold were used for further analysis in GeoGebra Classic 6 (GeoGebra GmbH, Linz, Austria) software. Twenty randomly chosen cells per image were marked with a line segment spanning the longest possible distance within the cell. To analyze MSC orientation, images obtained with the 4× objective were used to calculate the mean angular deviation from the median angle of cell orientation. Mean MSC length was calculated based on the measurements in images obtained with the 10× objective.

### 4.7. Quantitative RT-PCR

RNA was isolated using the RNeasy Mini Kit (Qiagen, Hilden, Germany) according to the manufacturer’s instructions, including an on-column DNAse treatment. Prior to isolation, cell aggregates and scaffolds were shredded in RLT buffer (RNeasy Mini Kit) containing 0.143 M β-mercaptoethanol using a TissueLyser II (Qiagen) at 30 Hz for 2 min and 2 × 2 min, respectively. A 2250 ng measure of total RNA was reverse transcribed using the RevertAid H Minus First Strand cDNA Synthesis Kit (Thermo Fisher). One microliter (corresponding to 37.5 ng RNA) of the obtained cDNA was used as template in each qPCR reaction. qPCR was performed employing the SYBR Green Mastermix (Bio-Rad, Hercules, CA, USA) on a qTowerG (Analytik Jena). The primers (Table 1) were defined using PrimerBlast (NCBI) and synthesized at IDT (Integrated DNA Technologies, Leuven, Belgium). PCR performance was checked by running a melting curve and a dilution series of pooled experimental cDNA to determine the efficiency of the reaction. Fold changes were calculated using the Pfaffl formula [55].

### 4.8. Supernatant Analyses

The soluble sulfated glycosaminoglycan content and total collagen content in the supernatants were measured using colorimetric assays (Glycosaminoglycan Assay Blyscan™, Carrickfergus, UK, and Sirius Red Total Collagen Detection Assay, Chondrex, Woodinville, WA, USA), the latter after concentrating the collagen content with polyethylene glycol solution (Chondrex). The assays were performed according to the manufacturer’s instructions. To determine MMP activity in the supernatants, a total MMP activity assay kit (Abcam, Cambridge, UK) was used according to the provided instructions, with an activation of MMPs for 3 h and fluorescence measurement 30 min after adding the fluorescent substrate. All measurements were performed using an Infinite^®^ M200 Pro plate reader (Tecan, Maennedorf, Switzerland).

### 4.9. Statistical Analysis

Statistical analysis was performed using IBM SPSS Statistics 28. For comparisons between groups, Mann–Whitney-U tests were used. For paired comparison of differences over time, Wilcoxon tests or Friedman tests with Wilcoxon post hoc tests and Bonferroni correction were performed. *p*-values < 0.05 were considered significant.

## 5. Conclusions

The current data showed that MSC matrix remodeling activity in MSCs is globally reduced under the early influence of chronically diseased tendon matrix. Given that at least some extent of matrix degradation and remodeling is crucial for therapeutic success when attempting to treat chronic tendon disease, this remains an issue that needs to be remediated. As the lower expression of MMPs in the tendon disease model was mainly observed on day 3 and recovered over time, there may be a chance to educate the MSCs in advance to prevent this effect.

## Figures and Tables

**Table 1:** Primer sequences used for qRT-PCR.

Gene	Primer Sequence	Accession No.	PCR Product (bp)
GAPDH	For: TGGAGAAAGCTGCCAAATACG Rev: GGCCTTTCTCCTTCTCTTGC	NM_001163856.1	309
COL1A2	For: CAACCGGAGATAGAGGACCA Rev: CAGGTCCTTGGAAACCTTGA	XM_001492939.3	243
COL3A1	For: AGGGGACCTGGTTACTGCTT Rev: TCTCTGGGTTGGGACAGTCT	XM_001917620.3	216
SCX	For: TACCTGGGTTTTCTTCTGGTCACT Rev: TATCAAAGACACAAGATGCCAGC	NM_001105150.1	51
TNC	For: TGAATATGGAATTGGAGTGTCTGC Rev: GGAGTTCTCCACACCAGAGTC	XM_023628743.1	147
DCN	For: GGCTGGCGGATCATAAGTACA Rev: CCAGGTGGGCAGAAGTCATT	NM_001081925.2	88
MKX	For: AAGATACTCTTGGCGCTCGG Rev: ACACTAAGCCGCTCAGCATT	XM_014737017.1	170
CTGF	For: CGACTGGAAGACACGTTTGG Rev: GTCTTGGAACAGGCACTCCA	XM_023651101.1	89
TGFB1	For: AATTCCTGGCGCTACCTCAG Rev: GAACTGAACCCGTTGATGCC	NM_001081849.1	194
TGFB3	For: TGCCCCAAAGGAATCACCTC Rev: GCGTTGTTTGGCGATATGCT	XM_001492687.6	186
MMP1	For: TGCCAAATGGACTTCAAGCTG Rev: GCCGAAGGATCTGTGGATGT	NM_001081847.2	137
MMP3	For: CACTAGCCATGTGCGATCCT Rev: GTCCTGAAGGTTTTGCGCC	NM_001082495.2	106
MMP9	For: GGCCAGTTCCAGACCTTTGA Rev: GGCAAGTCTCCCGAGTAGTTT	NM_001111302.1	86
MMP13	For: CACCTACACTGGCAAAAGCC Rev: TGGGATGTTTAGGGTTCGGG	NM_001081804.1	104
MMP14	For: GGCATCCAGCAACTTTACGG Rev: GCTTATCAGGGACAGAGGGC	XM_023621963.1	94
TIMP1	For: GTCTCCGGCATTCTGTTGTTG Rev: GCCCTGATGACGAACTCAGA	NM_001082515.1	110
TIMP2	For: AGTTCATCTACACGGCTCCC Rev: GCCTTTCCTGCGATGAGGTA	XM_023651899.1	88
TIMP3	For: AGGACTCTGCAACTTCGTGG Rev: GTCACGTAGCAAGGCAGGTA	NM_001081870.2	129
TIMP4	For: TTGAGTGCTTGGTGCAAAGTG Rev: TCTCCTTTCCCCAACTAAAGCA	XM_001492577.4	91

## Data Availability

The data presented in this study will be made available upon publication.

## Supplementary Materials

The following are available online at https://www.mdpi.com/article/10.3390/ijms222312798/s1. References [68,69] are cited in the supplementary materials.

## References

[1] Williams R.B., Harkins L.S., Hammond C.J., Wood J.L. (2001). Racehorse injuries, clinical problems and fatalities recorded on British racecourses from flat racing and National Hunt racing during 1996, 1997 and 1998. Equine Vet. J..

[2] Snedeker J.G., Foolen J. (2017). Tendon injury and repair-A perspective on the basic mechanisms of tendon disease and future clinical therapy. Acta Biomater..

[3] Dahlgren L., Mohammed H., Nixon A. (2005). Temporal expression of growth factors and matrix molecules in healing tendon lesions. J. Orthop. Res..

[4] Biernacka A., Dobaczewski M., Frangogiannis N.G. (2011). TGF-β signaling in fibrosis. Growth Factors.

[5] Jones G.C., Corps A.N., Pennington C.J., Clark I.M., Edwards D.R., Bradley M.M., Hazleman B.L., Riley G.P. (2006). Expression profiling of metalloproteinases and tissue inhibitors of metalloproteinases in normal and degenerate human achilles tendon. Arthritis Rheum..

[6] Patterson-Kane J.C., Becker D.L., Rich T. (2012). The pathogenesis of tendon microdamage in athletes: The horse as a natural model for basic cellular research. J. Comp. Pathol..

[7] Ely E., Avella C., Price J., Smith R., Wood J., Verheyen K. (2009). Descriptive epidemiology of fracture, tendon and suspensory ligament injuries in National Hunt racehorses in training. Equine Vet. J..

[8] Spaas J., Guest D., Van de Walle G. (2012). Tendon Regeneration in Human and Equine Athletes Ubi Sumus-Quo Vadimus (Where are We and Where are We Going to)?. Sports Med..

[9] Godwin E., Young N., Dudhia J., Beamish I., Smith R. (2011). Implantation of bone marrow-derived mesenchymal stemcells demonstrates improved outcome in horses with overstrain injury of the superficial digital flexor tendon. Equine Vet. J..

[10] Burk J., Brehm W. (2011). Stammzellentherapie von Sehnenverletzungen–klinische Ergebnisse von 98 Fällen [Stem cell therapy for tendon injuries-clinical results of 98 cases]. Pferdeheilkunde.

[11] Del Bue M., Riccò S., Ramoni R., Conti V., Gnudi G., Grolli S. (2008). Equine adipose-tissue derived mesenchymal stem cells and platelet concentrates: Their association in vitro and in vivo. Vet. Res. Commun..

[12] Smith R.K., Webbon P.M. (2005). Harnessing the stem cell for the treatment of tendon injuries: Heralding a new dawn?. Br. J. Sports Med..

[13] Burk J. (2019). Mechanisms of Action of Multipotent Mesenchymal Stromal Cells in Tendon Disease. Tendons.

[14] Wu Y., Peng Y., Gao D., Feng C., Yuan X., Li H., Wang Y., Yang L., Huang S., Fu X. (2015). Mesenchymal stem cells suppress fibroblast proliferation and reduce skin fibrosis through a TGF-β3-dependent activation. Int J. Low. Extrem. Wounds.

[15] Zhang L., Li Y., Guan C.Y., Tian S., Lv X.D., Li J.H., Ma X., Xia H.F. (2018). Therapeutic effect of human umbilical cord-derived mesenchymal stem cells on injured rat endometrium during its chronic phase. Stem Cell Res. Ther..

[16] Hiwatashi N., Bing R., Kraja I., Branski R. (2017). Mesenchymal stem cells have antifibrotic effects on transforming growth factor-β1-stimulated vocal fold fibroblasts. Laryngoscope.

[17] Lozito T., Jackson W., Nesti L., Tuan R. (2014). Human mesenchymal stem cells generate a distinct pericellular zone of MMP activities via binding of MMPs and secretion of high levels of TIMPs. Matrix Biol..

[18] Lozito T., Tuan R. (2011). Mesenchymal stem cells inhibit both endogenous and exogenous MMPs via secreted TIMPs. J. Cell Physiol..

[19] Roth S.P., Schubert S., Scheibe P., Groß C., Brehm W., Burk J. (2018). Growth Factor-Mediated Tenogenic Induction of Multipotent Mesenchymal Stromal Cells Is Altered by the Microenvironment of Tendon Matrix. Cell Transplant..

[20] Youngstrom D.W., Barrett J.G. (2016). Engineering Tendon: Scaffolds, Bioreactors, and Models of Regeneration. Stem Cells Int..

[21] Wang S., Wang Y., Song L., Chen J., Ma Y., Chen Y., Fan S., Su M., Lin X. (2017). Decellularized tendon as a prospective scaffold for tendon repair. Mater. Sci. Eng. C Mater. Biol. Appl..

[22] Samiric T., Parkinson J., Ilic M.Z., Cook J., Feller J.A., Handley C.J. (2009). Changes in the composition of the extracellular matrix in patellar tendinopathy. Matrix Biol..

[23] Berner D., Brehm W., Gerlach K., Offhaus J., Scharner D., Burk J. (2020). Variation in the MRI signal intensity of naturally occurring equine superficial digital flexor tendinopathies over a 12-month period. Vet. Rec..

[24] Baraniak P.R., McDevitt T.C. (2012). Scaffold-free culture of mesenchymal stem cell spheroids in suspension preserves multilineage potential. Cell Tissue Res..

[25] Beachley V., Kasyanov V., Nagy-Mehesz A., Norris R., Ozolanta I., Kalejs M., Stradins P., Baptista L., da Silva K., Grainjero J. (2014). The fusion of tissue spheroids attached to pre-stretched electrospun polyurethane scaffolds. J. Tissue Eng..

[26] Schwarz S., Gögele C., Ondruschka B., Hammer N., Kohl B., Schulze-Tanzil G. (2019). Migrating Myofibroblastic Iliotibial Band-Derived Fibroblasts Represent a Promising Cell Source for Ligament Reconstruction. Int. J. Mol. Sci..

[27] Schneider I., Baumgartner W., Groninger O., Stark W.J., Marsmann S., Calcagni M., Cinelli P., Wolint P., Buschmann J. (2020). 3D microtissue-derived human stem cells seeded on electrospun nanocomposites under shear stress: Modulation of gene expression. J. Mech. Behav. Biomed. Mater..

[28] Frith J.E., Thomson B., Genever P.G. (2010). Dynamic three-dimensional culture methods enhance mesenchymal stem cell properties and increase therapeutic potential. Tissue Eng. Part. C Methods.

[29] Schulze-Tanzil G., Mobasheri A., Clegg P.D., Sendzik J., John T., Shakibaei M. (2004). Cultivation of human tenocytes in high-density culture. Histochem. Cell Biol..

[30] Jauković A., Abadjieva D., Trivanović D., Stoyanova E., Kostadinova M., Pashova S., Kestendjieva S., Kukolj T., Jeseta M., Kistanova E. (2020). Specificity of 3D MSC Spheroids Microenvironment: Impact on MSC Behavior and Properties. Stem Cell Rev. Rep..

[31] Cheng N.C., Chen S.Y., Li J.R., Young T.H. (2013). Short-term spheroid formation enhances the regenerative capacity of adipose-derived stem cells by promoting stemness, angiogenesis, and chemotaxis. Stem Cells Transl. Med..

[32] Hsu S.H., Hsieh P.S. (2015). Self-assembled adult adipose-derived stem cell spheroids combined with biomaterials promote wound healing in a rat skin repair model. Wound Repair. Regen..

[33] Santos J.M., Camões S.P., Filipe E., Cipriano M., Barcia R.N., Filipe M., Teixeira M., Simões S., Gaspar M., Mosqueira D. (2015). Three-dimensional spheroid cell culture of umbilical cord tissue-derived mesenchymal stromal cells leads to enhanced paracrine induction of wound healing. Stem Cell Res. Ther..

[34] Redondo-Castro E., Cunningham C.J., Miller J., Brown H., Allan S.M., Pinteaux E. (2018). Changes in the secretome of tri-dimensional spheroid-cultured human mesenchymal stem cells in vitro by interleukin-1 priming. Stem Cell Res. Ther..

[35] Joshi J., Abnavi M.D., Kothapalli C.R. (2019). Synthesis and secretome release by human bone marrow mesenchymal stem cell spheroids within three-dimensional collagen hydrogels: Integrating experiments and modelling. J. Tissue Eng. Regen Med..

[36] Yao L., Bestwick C.S., Bestwick L.A., Maffulli N., Aspden R.M. (2006). Phenotypic drift in human tenocyte culture. Tissue Eng..

[37] Stoll C., John T., Endres M., Rosen C., Kaps C., Kohl B., Sittinger M., Ertel W., Schulze-Tanzil G. (2010). Extracellular matrix expression of human tenocytes in three-dimensional air-liquid and PLGA cultures compared with tendon tissue: Implications for tendon tissue engineering. J. Orthop. Res..

[38] Schneider P.R., Buhrmann C., Mobasheri A., Matis U., Shakibaei M. (2011). Three-dimensional high-density co-culture with primary tenocytes induces tenogenic differentiation in mesenchymal stem cells. J. Orthop. Res..

[39] Hahner J., Hoyer M., Hillig S., Schulze-Tanzil G., Meyer M., Schroepfer M., Lohan A., Garbe L.A., Heinrich G., Breier A. (2015). Diffusion chamber system for testing of collagen-based cell migration barriers for separation of ligament enthesis zones in tissue-engineered ACL constructs. J. Biomater. Sci. Polym. Ed..

[40] Hoyer M., Meier C., Breier A., Hahner J., Heinrich G., Drechsel N., Meyer M., Rentsch C., Garbe L.A., Ertel W. (2015). In vitro characterization of self-assembled anterior cruciate ligament cell spheroids for ligament tissue engineering. Histochem. Cell Biol..

[41] Laschke M.W., Menger M.D. (2017). Life is 3D: Boosting Spheroid Function for Tissue Engineering. Trends Biotechnol..

[42] Kuo C.K., Marturano J.E., Tuan R.S. (2010). Novel strategies in tendon and ligament tissue engineering: Advanced biomaterials and regeneration motifs. Sports Med. Arthrosc. Rehabil. Ther. Technol..

[43] Longo U.G., Lamberti A., Petrillo S., Maffulli N., Denaro V. (2012). Scaffolds in tendon tissue engineering. Stem Cells Int..

[44] Li X., Pongkitwitoon S., Lu H., Lee C., Gelberman R., Thomopoulos S. (2019). CTGF induces tenogenic differentiation and proliferation of adipose-derived stromal cells. J. Orthop. Res..

[45] Shen H., Jayaram R., Yoneda S., Linderman S.W., Sakiyama-Elbert S.E., Xia Y., Gelberman R.H., Thom Poulos S. (2018). The effect of adipose-derived stem cell sheets and CTGF on early flexor tendon healing in a canine model. Sci Rep..

[46] Ramazani Y., Knops N., Elmonem M.A., Nguyen T.Q., Arcolino F.O., van den Heuvel L., Levtchenko E., Kuypers D., Goldschmeding R. (2018). Connective tissue growth factor (CTGF) from basics to clinics. Matrix Biol..

[47] Riley G.P., Harrall R.L., Constant C.R., Chard M.D., Cawston T.E., Hazleman B.L. (1994). Tendon degeneration and chronic shoulder pain: Changes in the collagen composition of the human rotator cuff tendons in rotator cuff tendinitis. Ann. Rheum. Dis..

[48] Jacobson E., Dart A.J., Mondori T., Horadogoda N., Jeffcott L.B., Little C.B., Smith M.M. (2015). Focal experimental injury leads to widespread gene expression and histologic changes in equine flexor tendons. PLoS ONE.

[49] Kolb M., Margetts P.J., Sime P.J., Gauldie J. (2001). Proteoglycans decorin and biglycan differentially modulate TGF-beta-mediated fibrotic responses in the lung. Am. J. Physiol. Lung Cell Mol. Physiol..

[50] Hosaka Y., Kirisawa R., Mafune N., Takehana K. (2005). Downregulation of decorin and transforming growth factor-beta1 by decorin gene suppression in tendinocytes. Connect. Tissue Res..

[51] Delgado Caceres M., Pfeifer C.G., Docheva D. (2018). Understanding Tendons: Lessons from Transgenic Mouse Models. Stem Cells Dev..

[52] Jones E.R., Jones G.C., Legerlotz K., Riley G.P. (2013). Cyclical strain modulates metalloprotease and matrix gene expression in human tenocytes via activation of TGFβ. Biochim. Biophys. Acta.

[53] Yang G., Rothrauff B.B., Lin H., Gottardi R., Alexander P.G., Tuan R.S. (2013). Enhancement of tenogenic differentiation of human adipose stem cells by tendon-derived extracellular matrix. Biomaterials.

[54] Maeda E., Kuroyanagi K., Ando Y., Matsumoto T. (2020). Effects of Substrate Stiffness on Morphology and MMP-1 Gene Expression in Tenocytes Stimulated With Interleukin-1β. J. Orthop. Res..

[55] Pfaffl M.W. (2001). A new mathematical model for relative quantification in real-time RT-PCR. Nucleic Acids Res..

[56] Sassmann A., Lauermann A., Kasper C., Schubert S., Burk J. (2019). Context-sensitive extracellular matrix remodelling by human multipotent mesenchymal stromal cells. Acta Physiol..

[57] Minkwitz S., Schmock A., Kurtoglu A., Tsitsilonis S., Manegold S., Wildemann B., Klatte-Schulz F. (2017). Time-Dependent Alterations of MMPs, TIMPs and Tendon Structure in Human Achilles Tendons after Acute Rupture. Int. J. Mol. Sci..

[58] Nomura M., Hosaka Y., Kasashima Y., Ueda H., Takehana K., Kuwano A., Arai K. (2007). Active expression of matrix metalloproteinase-13 mRNA in the granulation tissue of equine superficial digital flexor tendinitis. J. Vet. Med. Sci..

[59] Riley G.P., Curry V., DeGroot J., van El B., Verzijl N., Hazleman B.L., Bank R.A. (2002). Matrix metalloproteinase activities and their relationship with collagen remodelling in tendon pathology. Matrix Biol..

[60] Kessler M.W., Barr J., Greenwald R., Lane L.B., Dines J.S., Dines D.M., Drakos M.C., Grande D.A., Chahine N.O. (2014). Enhancement of Achilles tendon repair mediated by matrix metalloproteinase inhibition via systemic administration of doxycycline. J. Orthop. Res..

[61] Davis M.E., Gumucio J.P., Sugg K.B., Bedi A., Mendias C.L. (2013). MMP inhibition as a potential method to augment the healing of skeletal muscle and tendon extracellular matrix. J. Appl. Physiol..

[62] Baldwin S.J., Kreplak L., Lee J.M. (2019). MMP-9 selectively cleaves non-D-banded material on collagen fibrils with discrete plasticity damage in mechanically-overloaded tendon. J. Mech. Behav. Biomed. Mater..

[63] Karousou E., Ronga M., Vigetti D., Passi A., Maffulli N. (2008). Collagens, proteoglycans, MMP-2, MMP-9 and TIMPs in human achilles tendon rupture. Clin. Orthop. Relat. Res..

[64] Sahin H., Tholema N., Petersen W., Raschke M.J., Stange R. (2012). Impaired biomechanical properties correlate with neoangiogenesis as well as VEGF and MMP-3 expression during rat patellar tendon healing. J. Orthop. Res..

[65] Smith R., Werling N., Dakin S., Alam R., Goodship A., Dudhia J. (2013). Beneficial effects of autologous bone marrow-derived mesenchymal stem cells in naturally occurring tendinopathy. PLoS ONE.

[66] Romero A., Barrachina L., Ranera B., Remacha A., Moreno B., Blas I. (2017). Comparison of autologous bone marrow and adipose tissue derived mesenchymal stem cells, and platelet rich plasma, for treating surgically induced lesions of the equine superficial digital flexor tendon. Vet. J..

[67] Burk J., Erbe I., Berner D., Kacza J., Kasper C., Pfeiffer B., Winter K., Brehm W. (2014). Freeze-thaw cycles enhance decellularization of large tendons. Tissue Eng. Part. C Methods.

[68] Paebst F., Piehler D., Brehm W., Heller S., Schroeck C., Tárnok A., Burk J. (2014). Comparative immunophenotyping of equine multipotent mesenchymal stromal cells: An approach toward a standardized definition. Cytom. A.

[69] Hagen A., Lehmann H., Aurich S., Bauer N., Melzer M., Moellerberndt J., Patané V., Schnabel C.L., Burk J. (2021). Scalable Production of Equine Platelet Lysate for Multipotent Mesenchymal Stromal Cell Culture. Front. Bioeng. Biotechnol..

