# Supplementary material for: Mesenchymal Stromal Cells Adapt to Chronic Tendon Disease Environment with an Initial Reduction in Matrix Remodeling
Source: Int J Mol Sci. 2021 Nov 26;22(23):12798. doi: 10.3390/ijms222312798 (PMC8657831; doi:10.3390/ijms222312798)
Supplement: Supplementary file 1 [file ijms-22-12798-s001.zip › ijms-1466560-supplementary.pdf]

Supplement S1, Doll et al.: MSC characterization by trilineage differentiation and flow cytometry.

Trilineage Differentiation

The differentiation assays were performed with passage 4 MSC.

For adipogenic differentiation, 3,000 MSC per well were seeded in a 24 well plate and pre-incubated in standard culture medium. At day 4, medium was replaced by StemPro™ adipogenic differentiation medium (Gibco®, ThermoFisher Scientific, Darmstadt, Germany) supplemented with 0.1% gentamycin and 5% rabbit serum. After 3 days of differentiation, cells were fixed with 50% ethanol for 20 min and stained using Oil Red O for lipid vacuoles and hematoxylin counterstain.

For chondrogenic differentiation, 500,000 MSC per 15 ml tube were washed in PBS and StemPro™ chondrogenic differentiation medium (Gibco®) supplemented with 0.1% gentamycin was added. Tubes were centrifuged at 280 g for 5 min to form pellets and medium was changed every three days. On day 21, pellets were fixed with 4% paraformaldehyde for 12 h and subsequently embedded in paraffin. 5 µm sections were prepared and stained with Alcian blue.

For osteogenic differentiation, 2,000 MSC per well were seeded in a 24 well plate and pre-incubated in standard culture medium. On day 4, medium was replaced by StemPro™ osteogenic differentiation medium (Gibco®) supplemented with 0.1% gentamycin and changed every three days. After 21 days of differentiation, cells were fixed with 4% paraformaldehyde for 10 min and stained using von Kossa staining for extracellular mineralization.

MSC stained positive for adipogenic, chondrogenic and osteogenic differentiation (Figure S1).

Oil Red O **Alcian blue** **von Kossa**

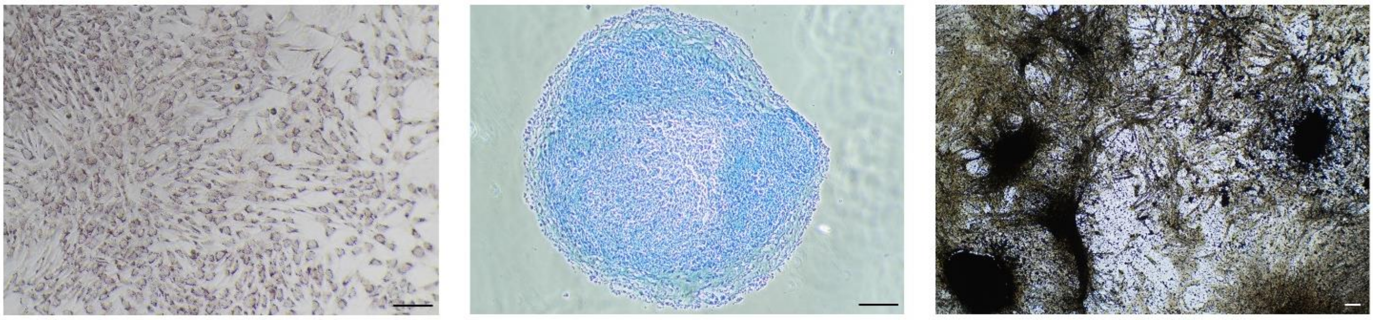

Figure S1: Brightfield images after differentiation and respective stainings. Oil Red O: adipogenic differentiation, Alcian blue: chondrogenic differentiation, von Kossa: osteogenic differentiation. Scale bars = 100 µm.

Supplement S1, Doll et al.: MSC characterization by trilineage differentiation and flow cytometry.

Flow Cytometry

MSC in passage 3 were used for analysis of surface antigens. 2 x 10^5 cells per sample were stained with monoclonal antibodies or corresponding isotype controls [68,69]. The exclusion of dead cells was achieved by staining with BD Horizon™ Fixable Viability Stain 620 (BD, Franklin Lakes, New Jersey, USA). Data acquisition and analysis was done on an AccuriC6 Plus flow cytometer (BD).

MSC were positive for CD29, CD44 and CD90 and partly positive for CD34; they were negative for CD105, CD14, CD45 and MHCII (Figure S2).

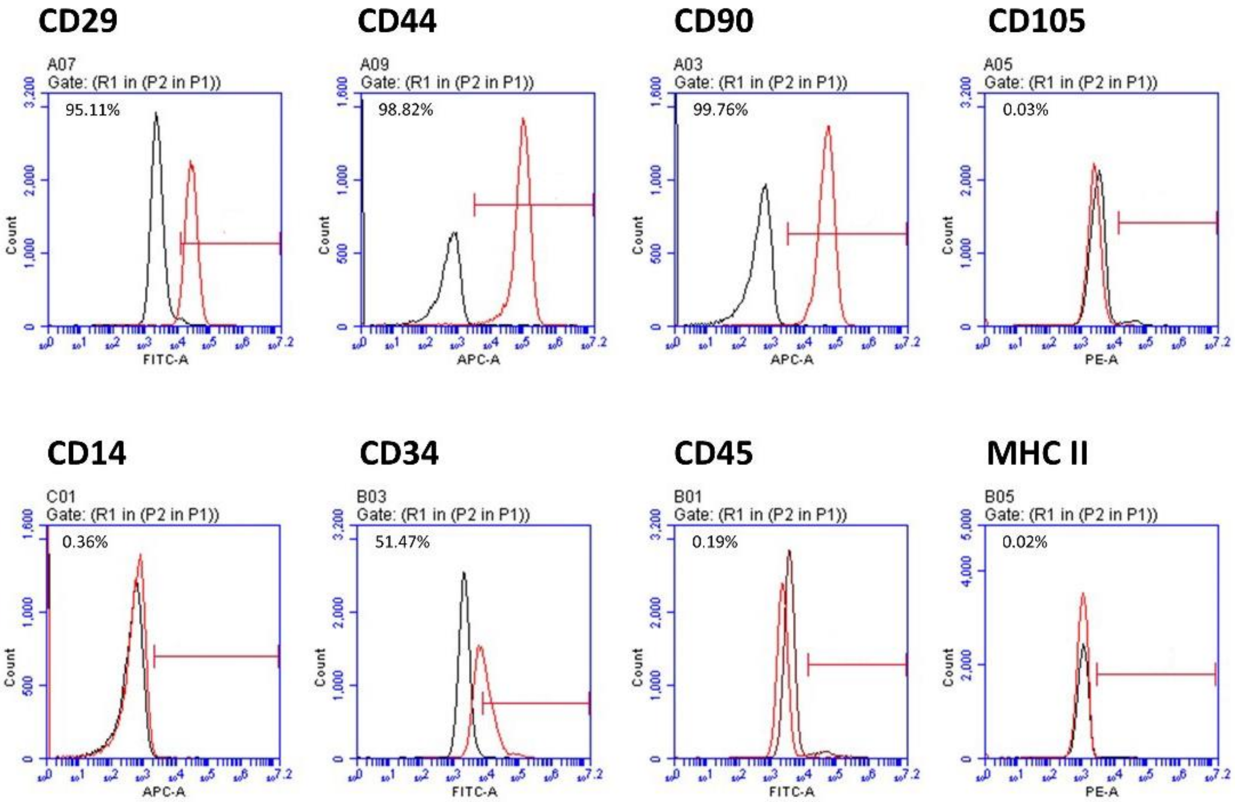

Figure S2: Flow cytometry analysis after mechanical cell detachment and surface antigen staining. Histograms show the number of events versus fluorescence intensity. Red shows the surface antigen staining with monoclonal antibodies and black the respective isotype control.
